# *De novo* identification and targeted sequencing of SSRs efficiently fingerprints *Sorghum bicolor* sub-population identity

**DOI:** 10.1371/journal.pone.0248213

**Published:** 2021-03-08

**Authors:** John P. Baggett, Richard L. Tillett, Elizabeth A. Cooper, Melinda K. Yerka

**Affiliations:** 1 Department of Biochemistry and Molecular Biology, University of Nevada, Reno, NV, United States of America; 2 Nevada Center for Bioinformatics, University of Nevada, Reno, NV, United States of America; 3 Department of Bioinformatics and Genomics, University of North Carolina at Charlotte, Charlotte, NC, United States of America; 4 Department of Agriculture, Veterinary, and Rangeland Sciences, University of Nevada, Reno, NV, United States of America; USDA-ARS Southern Regional Research Center, UNITED STATES

## Abstract

Recent plant breeding studies of several species have demonstrated the utility of combining molecular assessments of genetic distance into trait-linked SNP genotyping during the development of parent lines to maximize yield gains due to heterosis. SSRs (Short Sequence Repeats) are the molecular marker of choice to determine genetic diversity, but the methods historically used to sequence them have been burdensome. The ability to analyze SSRs in a higher-throughput manner independent of laboratory conditions would increase their utility in molecular ecology, germplasm curation, and plant breeding programs worldwide. This project reports simple bioinformatics methods that can be used to generate genome-wide *de novo* SSRs *in silico* followed by targeted Next Generation Sequencing (NGS) validation of those that provide the most information about sub-population identity of a breeding line, which influences heterotic group selection. While these methods were optimized in sorghum [*Sorghum bicolor* (L.) Moench], they were developed to be applied to any species with a reference genome and high-coverage whole-genome sequencing data of individuals from the sub-populations to be characterized. An analysis of published sorghum genomes selected to represent its five main races (bicolor, caudatum, durra, kafir, and guinea; 75 accessions total) identified 130,120 SSR motifs. Average lengths were 23.8 bp and 95% were between 10 and 92 bp, making them suitable for NGS. Validation through targeted sequencing amplified 188 of 192 assayed SSR loci. Results highlighted the distinctness of accessions from the guinea sub-group margaritiferum from all other sorghum accessions, consistent with previous studies of nuclear and mitochondrial DNA. SSRs that efficiently fingerprinted margaritiferum individuals (*Xgma1 –Xgma6*) are presented. Developing similar fingerprints of other sub-populations (*Xunr1 –Xunr182*) was not possible due to the extensive admixture between them in the data set analyzed. In summary, these methods were able to fingerprint specific sub-populations when rates of admixture between them are low.

## Introduction

Originating in Africa, sorghum [*Sorghum bicolor* (L.) Moench] (2*n* = 2*x* = 20) is a drought-tolerant C_4_ grass species having wide genetic diversity [1]. The fifth-most produced grain crop based on tonnage in the world, sorghum is used for biofuels, livestock feed, and human consumption, particularly in hot, dry regions of the developing world [2]. It is hypothesized that humans have been cultivating sorghum for >8,000 years [3]. Ancient farmers carried sorghum seeds throughout Africa and eventually to India and China, adapting germplasm to each new environment as they went, resulting in the diversity seen today. The five main cultivated races of sorghum (bicolor, caudatum, durra, guinea, and kafir) and working groups within them are typically identified based on panicle architecture and seed morphology [4]. There is an increasing amount of genomic sequence data available in sorghum that can be used to construct SNP-based haplotypes to differentiate between races and working groups (collectively referred to as sub-populations through this manuscript) at the genomic level, but this has yet to be leveraged efficiently in the development of molecular markers that could be used by breeders. For example, a working group within guinea, known as margaritiferum, has been shown to be distinct from the rest of the guinea race by both mitochondrial [5] and genomic [6–8] DNA analyses, and contains a large amount of untapped genetic diversity [9]. This diversity notably includes many rare alleles [8], as well as desirable traits such as high nitrogen use efficiency [10] and aluminum tolerance [11]. Currently, the identification of margaritiferum from other guinea accessions is based on differences in seed size, as set forth by Harlan and de Wet in 1972 [4]. However, seed size in sorghum is a variable phenotype that is strongly influenced by both high [12,13] and low [14] temperatures during reproductive development and the growth environment [15], making phenotypic identification difficult. Recent studies [16,17] have emphasized the need for better molecular markers than SNP haplotypes to more accurately and consistently assign accessions to a race or working group to distinguish among sub-populations of sorghum and assist in developing better heterotic groups.

Short Sequence Repeats (SSRs), also known as microsatellites and short tandem repeats, are the marker of choice for determining genetic diversity due to their high number of alleles per locus and mutation frequency [18–20]. Hypervariability in the quantity of repeated motifs [21] results in higher levels of polymorphism and mutation rates than single nucleotide polymorphisms (SNPs) and provides more information per location sequenced [18]. SSRs can be quickly and inexpensively screened through PCR amplification followed by gel separation. For these reasons, SSRs have been used for many years by plant geneticists, ecologists, and breeders. However, with the advent of next-generation sequencing (NGS) methods in the early 2000s, researchers quickly switched to using single nucleotide polymorphisms (SNPs) from whole-genome sequencing (WGS) and genotyping-by-sequencing (GBS) data sets to replace SSRs because NGS methods are much higher-throughput and, being digital, the data can be stored indefinitely and do not vary across laboratory conditions.

Following the original release of the *Sorghum bicolor* reference genome in 2009 [22], publications have emerged to make WGS and GBS data publicly available for sorghum germplasm [1,23–25]. SNPs from these data sets have been successfully used to construct haplotypes for the analysis of genetic diversity [23,24,26] and, in conjunction with phenotypic data [27–29], to make gene-trait associations for breeding using genomic selection (GS). However, despite these advancements in the throughput of sorghum genetics work, much remains unknown about the molecular basis of heterosis, which derives from population structure within the species. Theoretically, SSR loci would provide better information about population structure than a comparable number of SNP loci [18], but they come with the limitations outlined above. In addition, with all of the published SSRs in sorghum [7,30–37], their primer sites are not amenable to targeted NGS and they were not developed specifically for their ability to differentiate sub-populations, making *de novo* identification and NGS validation necessary.

Rapid and consistent screening for population structure could be achieved if SSRs that accurately assign sorghum germplasm to races and working groups were optimized for newer and less expensive NGS methods like targeted sequencing. Currently, the volume of publicly available WGS data provides the ideal platform for *in silico* identification of SSR loci to achieve much higher genome coverage than was previously available for the best possible genetic diversity characterizations. Conventional wisdom has held that SSRs may be too long for reliable targeted sequencing due to amplicon length restrictions. In recent years, this has been proven untrue as NGS methodologies for sequencing SSRs have worked in multiple species, such as rice (*Oryza sativa* L.), cucumber (*Cucumis sativus* L.) and golden pompano (*Trachinotus ovatus* Linnaeus, 1758) [38–41]. While these studies showed the utility of NGS for large scale SSR sequencing, their focus was on the creation of new methodologies, technologies, and pipelines rather than routine screening for sub-population assignments. While such information is useful for discovery genetics purposes, a commercially-available panel of SSRs that is accessible to all breeders (including those lacking a molecular lab and NGS capabilities) would be vastly more efficient at standardizing sub-population assignments of sorghum accessions around the world. Custom panels of SSRs identified in this work can be sequenced using a variety of targeted sequencing methods at commercial labs that can also extract DNA and assist with bioinformatics.

Genetic diversity information from Diversity Arrays Technology (DArT) markers has been used in wheat [*Triticum aestivum* L.] to assist in the selection of parent lines having greater genetic distances among them to achieve improved heterosis in grain yield [42]. Jaccard genetic distance coefficients (*d*) among wheat parents in the study ranged from 0 to 0.76, with an overall mean of 0.55. Thus, giving a Jaccard’s similarity coefficient of 1 to .24 with an overall mean of .45. In maize [*Zea mays* L.], heterosis observed in grain yield and most yield components was positively correlated with greater genetic distance among parents whose % relatedness ranged from 0.18 to 0.33, as determined by SNP and SilicoDArT markers [43]. Similar studies have been conducted in sorghum over the years using increasingly advanced DNA sequencing technologies. Jordan et al. (2003) [44] used RFLP markers and Mindaye et al. (2016) [45] used SSR markers; both studies reported a positive correlation between genetic distance among sorghum parents and heterosis for grain yield. Conversely, no correlation between genetic distance and heterosis was observed by Amelework et al. (2017) [46], who measured genetic distance with phenotypic and SSR markers; or by Crozier et al. (2020) [16], who used GBS-SNP data. Crozier et al. (2020) reported that genetic similarity among elite grain sorghum parents in the U.S. ranged from 0.63 to 0.79, which is quite high compared to the afore-mentioned wheat and maize studies. The authors noted that the lack of correlation could be due to the decreased genetic diversity information that results from reduced-complexity genotyping or the relatively high degree of relatedness among elite male and female parents in this crop. Either way, improved genotyping methods that efficiently and inexpensively assess population structure could be used in breeding programs to broaden the genetic distance among elite hybrid parents of sorghum to better test for and exploit heterosis in traits related to grain, biomass, or sugar yield [16,17].

The current study conducted a re-analysis of sorghum accessions sequenced from various published works [1,22,25] as a proof of concept that SSRs could be *de novo* identified from WGS data and optimized for targeted sequencing and population structure determination, including the distinction of races and working groups. To the best of the authors’ knowledge, this is the first use of version three of the *Sorghum bicolor* genome assembly, which was assembled using long reads (~30 kb) as opposed to previous versions assembled from short reads (resulting in less ambiguity within repetitive regions where SSRs reside), to identify sub-population-specific SSRs at much wider genome coverage. The methods outlined herein demonstrate that large sets of SSRs can be optimized for targeted sequencing to easily analyze many different genomic locations during single runs. These runs are easily compiled into Microsoft Excel or CSV files that are accessible to those with limited bioinformatics training. This helps breeders to better understand and utilize the genetic diversity within their breeding populations without the need for expensive specialized equipment.

The aim of this project was the *de novo* identification of SSRs for sub-population determination in sorghum. This work provides the first publicly available resource to genetically differentiate sub-populations using targeted sequencing methods that are more economical than genome-wide SNPs and that also provide a more accurate picture of genetic diversity. As a proof of concept, this work identified NGS-validated SSRs that successfully differentiated the guinea working group, margaritiferum, (*Xgma1 –Xgma6*), from the five main races of sorghum. This work also provides an additional 182 NGS-validated SSRs for analyzing genetic diversity (*Xunr1 –Xunr182*). All SSRs are presented with their genomic locations and suitable primers for their extension using either gel-based or NGS techniques. Once additional WGS data is published that evenly and comprehensively represents the five main races, similar fingerprints for each of them will also be possible. Finally, these methods can be deployed in any species with a reference genome to assist molecular ecology, germplasm curation, or conservation programs.

## Materials and methods

### Published sequencing data processing and SSR identification

The raw sequencing data of 75 sorghum accessions [1,22,25] were used for *in silico* analysis, and were obtained from the sequence read archive at NCBI. To distinguish samples by source, accessions from the first published data were named as published [22], while data from subsequent published works had suffix labels added to the accession’s names [1,25]. The suffixes added were: _TAMU, _UQ, _BGI, for data generated at Texas A&M University, University of Queensland, and BGI respectively. BWA (version: bwa-0.7.17) [47] was used with default settings to align to the *Sorghum bicolor* reference genome (Sbicolor_454_v3.0.1) [25]. GATK (version: gatk-4.0.5.1) [48] was used to identify variations and call haplotyes with the CreateSequenceDictionary, IndexFeatureFile, HaplotypeCaller, CombineGVCFs, and MergeVcfs tools. Subsequently, htslib (version: 1.8) in Samtools (version: 1.9) [49] was used with the subtool tabix to create an indexed file. Variant sites were then filtered prior to *de novo* SSR identification using PLINK software (version: 1.9) [50] with settings --mind 0.7 --maf 0.05 --geno 0.01 --allow-extra-chr --indep-pairwise 50 10 0.1 --double-id --vcf-inspace-to “_”. Filtered variants were processed with HipSTR (version: HipSTR-v0.6.2) [51] for SSR identification using the settings --min-reads 25 --no-rmdup --max-mate-dist 1000 --max-str-len 500 --max-reads 200 --def-stutter-model --require-pair.

### Genetic analysis of populations

For the genetic analysis of sorghum populations, fastSTRUCTURE [52] (version: fastSTRUCTURE-e47212f) was used to map the genetic structure at *K* = 2–10 (--full --seed = 100 --prior = logistic). Calculation of the log-marginal likelihood lower bound (LLBO) was performed in fastSTRUCTURE to determine the optimal value *K* within the population of study [52]. Plots were generated using the R [53] package ggplot2 (version: 3.2.1) [54] in RStudio [55]. SplitsTree4 (version: 4.15.1) [56] was used to create a split network tree. Colors were overlaid to the separate clades (putatively corresponding to races) of the tree based on the populations determined in fastSTRUCTURE [52].

### Venn diagram

SSR locations for each of the six populations (bicolor (*n* = 4), caudatum (*n* = 29), durra (*n* = 19, guinea (*n* = 3), kafir (*n* = 16), and margaritiferum (*n* = 4)) were assigned a score of 1 if there was a reference or alternative allele found in the previously published sequencing data [1,22,25] within each population. SSR locations were given a 0 if there was no reference or alternative allele found at that position of the chromosome in the genotype data of the Variant Call Format (VCF) file. Shared and unique sets of SSR candidates were visualized in R [53] using the package venn (version: 1.7) [57] in RStudio [55] with clades again colored to match those in fastSTRUCTURE [52] and SplitsTree4 [56].

### SSR filtering

The DNA sequences for sorghum sub-populations were compiled together and separated into VCF files arranged by chromosome. Through vcftools (version: 0.1.16) [58] VCF files were first filtered for allele quantity (--max-alleles 13 --min-alleles 2 --max-missing 1 --recode), then the vcftools function --singletons was used. Only the 4,179 unique doubletons, locations with a minor allele occurring in only one population homozygous for that allele, were taken from these files for identification of SSRs. Locations were targeted for primer design by LGC Genomics (Teddington, United Kingdom) using proprietary methods. Only locations with high specificity (no off-target sites throughout the genome for both forward and reverse primers) were considered beyond this stage. Reference and alternative alleles were used to calculate the standard deviation of SSR lengths between the populations in R [53]. SSRs with standard deviations above seven were retained for further filtering to focus on locations with maximal diversification within the population. SSRs within 1 Mb of the ends of each chromosome were filtered out to avoid telomeric regions. SSRs with a length less than 13 or greater than 49 were excluded. Finally, all SSRs with a motif length of one were removed. Based upon these criteria, 192 SSRs were validated using NGS sequencing.

### Plant material, DNA extraction, and sequencing

A total of 75 sorghum WGS data sets were published with sufficient sequencing depth for analysis at the time of the study [1,22,25], but a subset of only 53 of these accessions were available through the United States Department of Agriculture U.S National Plant Germplasm System Grin-Global [59] for validation of the new SSRs. Seedlings of these 53 accessions were grown in Pro-Mix® Biofungicide™ growing medium (Premier Tech Horticulture, Rivière-du-Loup, Qc) until they were large enough to provide leaf samples. Leaf discs were harvested from immature tissues using the LGC BioArk Leaf kits according to the manufacturer’s instructions. The DNA was extracted by LGC genomics using LGC sbeadex™ chemistry and libraries were prepared using the LGC genomics SeqSNP pipeline. Sequencing was performed by LGC genomics using Illumina® [60] sequencing-by-synthesis technology on an Illumina® NextSeq 550 with paired-end 150-base pair reads. All sequencing data is publicly available through the submission to NCBI (https://www.ncbi.nlm.nih.gov/) as BioProject (PRJNA610844), with 53 BioSample accession numbers (SAMN14318439—SAMN14318491), and 53 Sequence Read Archive (SRA) submission accession numbers (SRR11252447—SRR11252499).

### *In silico* SSR chromosomal mapping

*In silico* SSR chromosome mapping was performed using the R [53] package chromPlot (version: 1.12.0) [61] in RStudio [55]. SSRs were mapped using base settings except for the following: names were plotted at chromosomal location with the “stat” argument set to the SSR labels and the “noHist” argument used to avoid plotting histograms onto the output plots.

### Population genetics statistics

The genetic information statistics for polymorphism information content (PIC) and heterozygosity (H) [62] were calculated in Excel using the following equations:

$$H=1-\sum_{i=1}^{l}P_{i}^{2}$$

$$PIC=1-\sum_{i=1}^{l}P_{i}^{2}-\sum_{i=1}^{l-1}\sum_{j=i+1}^{l}2P_{i}^{2}P_{j}^{2}$$

Wherein *l* is the number of alleles in the locus and *P_i_* and *P_j_* are the allele frequencies of the i^th^ and j^th^ alleles, respectively.

## Results

**Fig 1:**
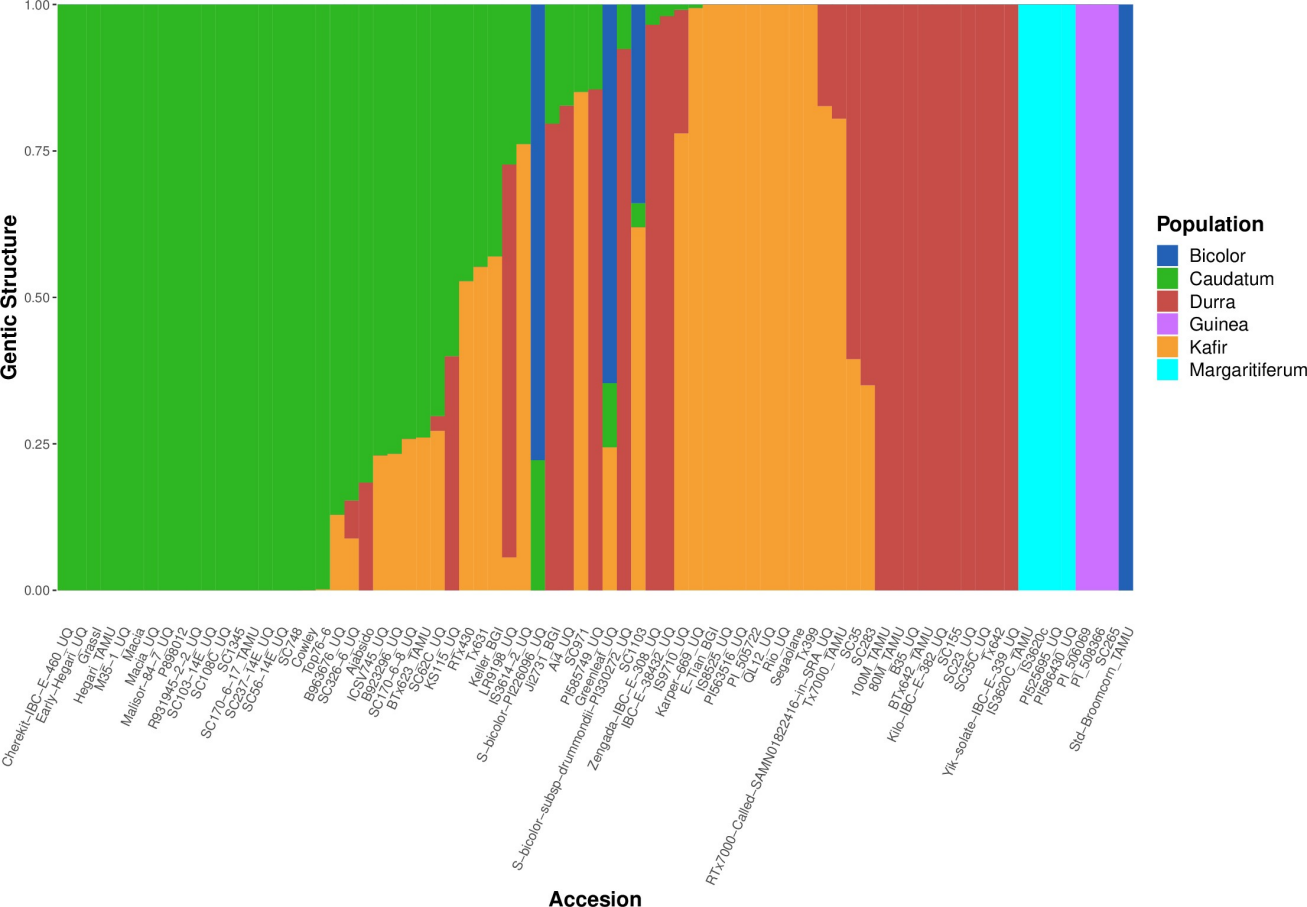
Six distinct sorghum sub-population distributions predicted by optimal fastSTRUCTURE grouping. fastSTRUCTURE mapping of the genetic structure of 75 sorghum accessions with optimized *K* = 6. The six sub-populations are depicted by: dark blue, green, red, purple, orange, and light blue representing bicolor, caudatum, durra, guinea, kafir, and margaritiferum respectively. The *x-axis* represents each accession and the *y-axis* represents the proportion of the genetic structure from the sorghum sub-populations.

**Fig 2:**
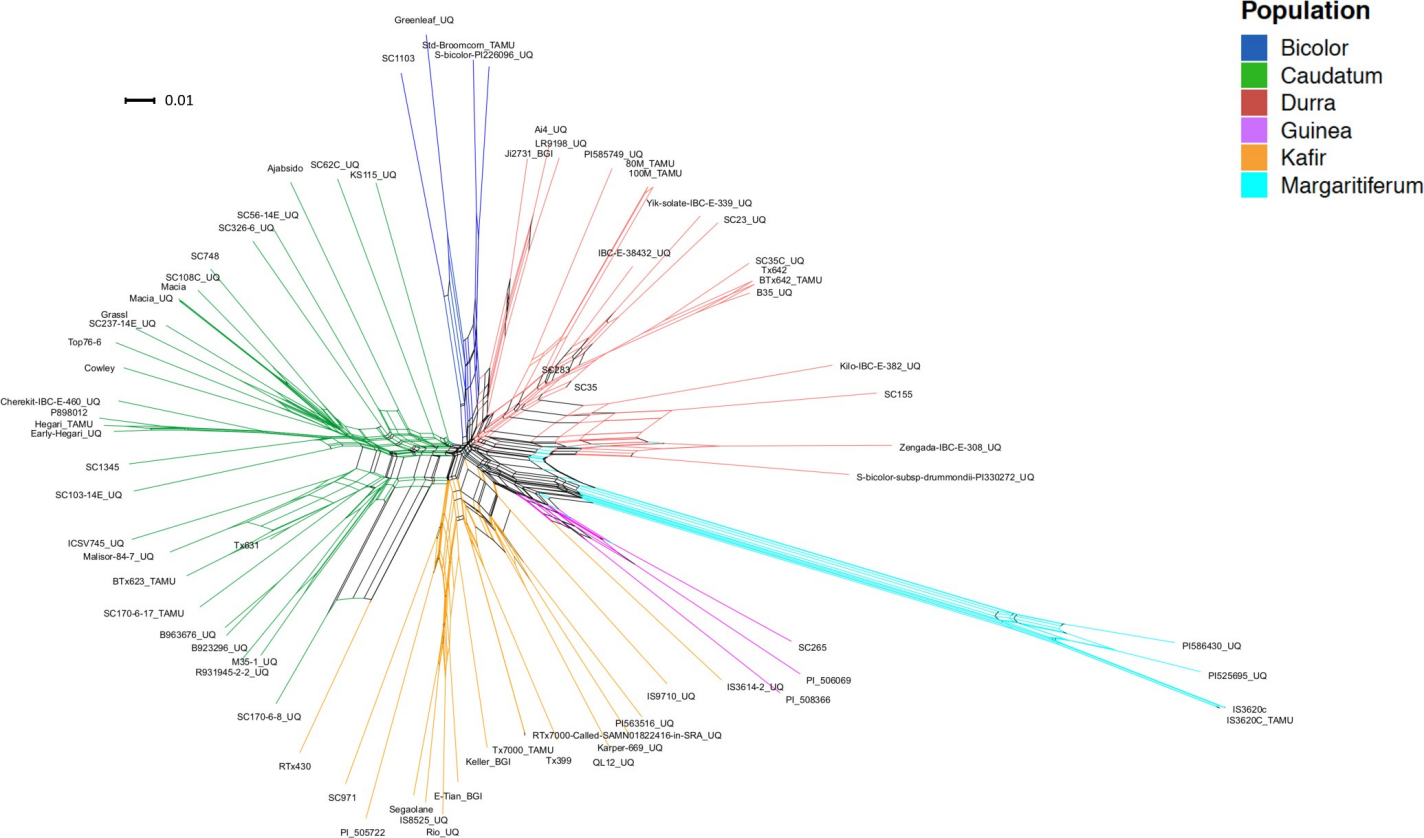
Split network divides sorghum into six clades. Split network diagram from SplitsTree4 depicting the genetic separation of 75 sorghum accessions. Splits-identified genetic divergence between accessions. Colors indicate the clade each accession belongs to. Dark blue, green, red, purple, orange, and light blue represent bicolor, caudatum, durra, guinea, kafir, and margaritiferum, respectively. Each node is labeled with the accession it represents. The scale bar is representative of a weight of 0.01 of the corresponding split.

In order to investigate genetic diversity in sorghum, a re-analysis was performed of WGS data from 75 published accessions [1,22,25], generating a map of 19,230,634 variants at 18,299,015 sites across the *S*. *bicolor* (Sbicolor_454_v3.0.1) genome [25]. Populations within the 75 samples were predicted using whole genome polymorphism data through fastSTRUCTURE for multiple *K* values, 2–10 (S1 Fig). By investigating the population size that maximizes the marginal likelihood through the LLBO curve (S2 Fig), the optimum population number *K* = 6 was found. Plotting *K* values two through ten (S1 Fig) demonstrated fastSTRUCTURE’s ability to avoid overfitting: *K* = 7–10 would not produce more than six populations with the seventh through tenth populations providing zero contribution to the overall genetic structure (S1 Fig). The use of fastSTRUCTURE with *K* = 6 (Fig 1) showed a distinct population genetic structure. The separation of the accessions into six populations further partitioned margaritiferum into a population distinct from guinea (Fig 1). At *K* = 2 (the first separation among sorghum populations), the genetic structure analysis depicts the margaritiferum accessions as different from the majority of accessions within the other populations (S1 Fig), indicating its genetic differentiation from all of the main races. The margaritiferum accessions remained distinct from *K* = 2–10 (S1 Fig). These findings are consistent with those of other researchers [1,5–8,63] indicating that margaritiferum accessions are distinct from guinea accessions. Independently of, and concurrent with fastSTRUCTURE (Figs 1, S1 and S2), split network analysis with SplitsTree4 also separated the 75 sorghum accessions into six clades using aligned WGS data for each samples [1,22,25] (Fig 2). The spatially-segregated accessions within the split network (Fig 2) again showed a sharp separation of accessions belonging to margaritiferum from guinea accessions, and the margaritiferum clade was the most distinct among all clades.

**Fig 3:**
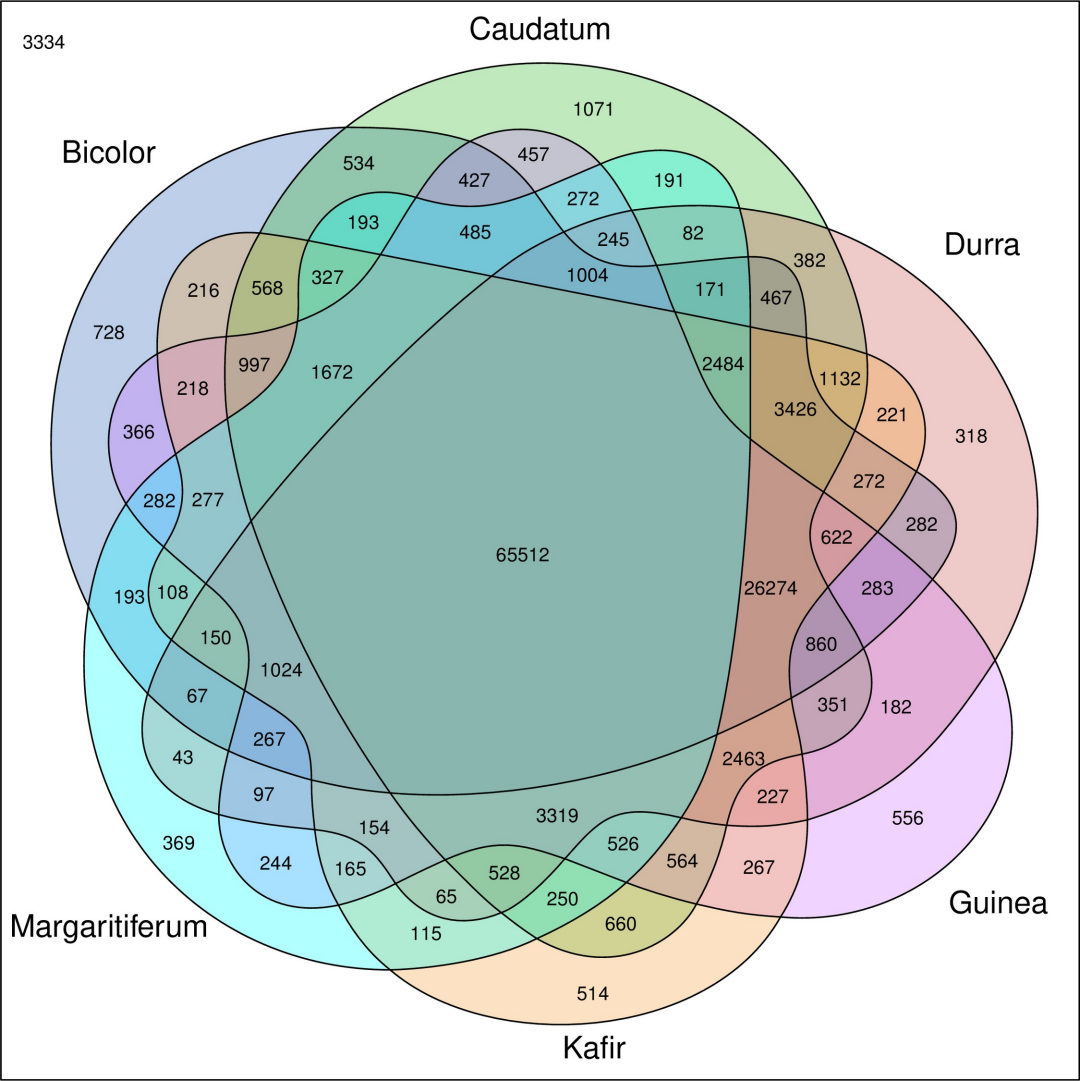
Venn diagram of *de novo* SSRs shows distinct SSRs for each clade. SSRs that are specific to only one clade in the merged VCF file are depicted by sections with no overlapping colors. SSRs that are shared by different clades are depicted within overlapping sectors. The SSRs that were identified in the *Sorghum bicolor* reference genome but had no reference or alternative alleles in the accessions evaluated by this study are denoted in the top left corner. The colored Venn regions are labeled with the sub-population they depict. Dark blue, green, red, purple, orange, and light blue represent bicolor (*n* = 4), caudatum (*n* = 29), durra (*n* = 19), guinea (*n* = 3), kafir (*n* = 16), and margaritiferum (*n* = 4), respectively.

*In silico* analysis of the *Sorghum bicolor* reference genome predicted 163,943 SSRs, of which 130,120 had mapped sequence data for the 75 accessions analyzed. Of the 130,120 SSRs identified, 18,080, 15,767, 15,857, 13,768, 11,392, 11,496, 11,255, 10,161, 10,895, and 11,449 were located on chromosomes one, two, three, four, five, six, seven, eight, nine, and ten respectively. In the merged VCF files, the majority showed allele presence/absence variation based on sub-population (Fig 3). While many SSR alleles were shared (65,512) between all six sub-populations, each one had at least 300+ SSR alleles that were unique to that sub-population (Fig 3). These unique SSR alleles highlight the divergence that has occurred between the main races of sorghum as well as the guinea working group margaritiferum, and the genetic diversity that is available to plant breeders. The key innovation in these methods was in filtering for doubletons–SSR loci that were unique to one sub-population and every individual in that sub-population was homozygous for that allele. This filtering method maximized the information obtained by the sequencing of each SSR so that more accurate population structure could be determined.

After filtering for doubletons, 4,179 sites passed with 516, 506, 558, 409, 397, 326, 458, 247, 311, and 451 SSRs located on Chromosomes one, two, three, four, five, six, seven, eight, nine, and ten, respectively. Doubleton filtering increased the likelihood of finding SSR alleles that were specific to only one sub-population upon sequencing. Filtering based on allele quantity removed SSRs with too few alleles to be informative or SSRs at sites that were too prone to mutation, which would decrease the heritability of SSR lengths and hence their ability to accurately track the sub-population identity of a breeding line across generations. Filtering out telomeric regions was important to prevent SSRs from being drawn from these gene-rich sites of high recombination and crossing over events [64,65] in the *Sorghum bicolor* genome. Unequal crossing over events along with replication strand slippage are the two main models for SSR length mutation [66]. The goal of the study was not to develop trait-linked SSRs in gene-rich regions of the genome, but to identify SSRs that stably, over many generations, assess genetic distance among sub-populations. This necessitated finding SSRs that mutate less frequently.

The 4,179 doubleton sites were targeted for primer design by LGC Genomics using proprietary methods. Final filtering for sites with high primer specificity, no off-target primer binding, and fragment length identified 192 SSRs that were selected for validation using targeted NGS. SSR length filtering is necessary to identify sites where the entire sequence of the SSR can be ascertained while taking into account the primer length (40 bp) and read lengths (150 bp) used. Selected SSRs were sequenced using Illumina technology [60] in all 53 publicly-available accessions from the USDA Grin-Global [59] out of the original 75 used for *in silico* analysis.

**Fig 4:**
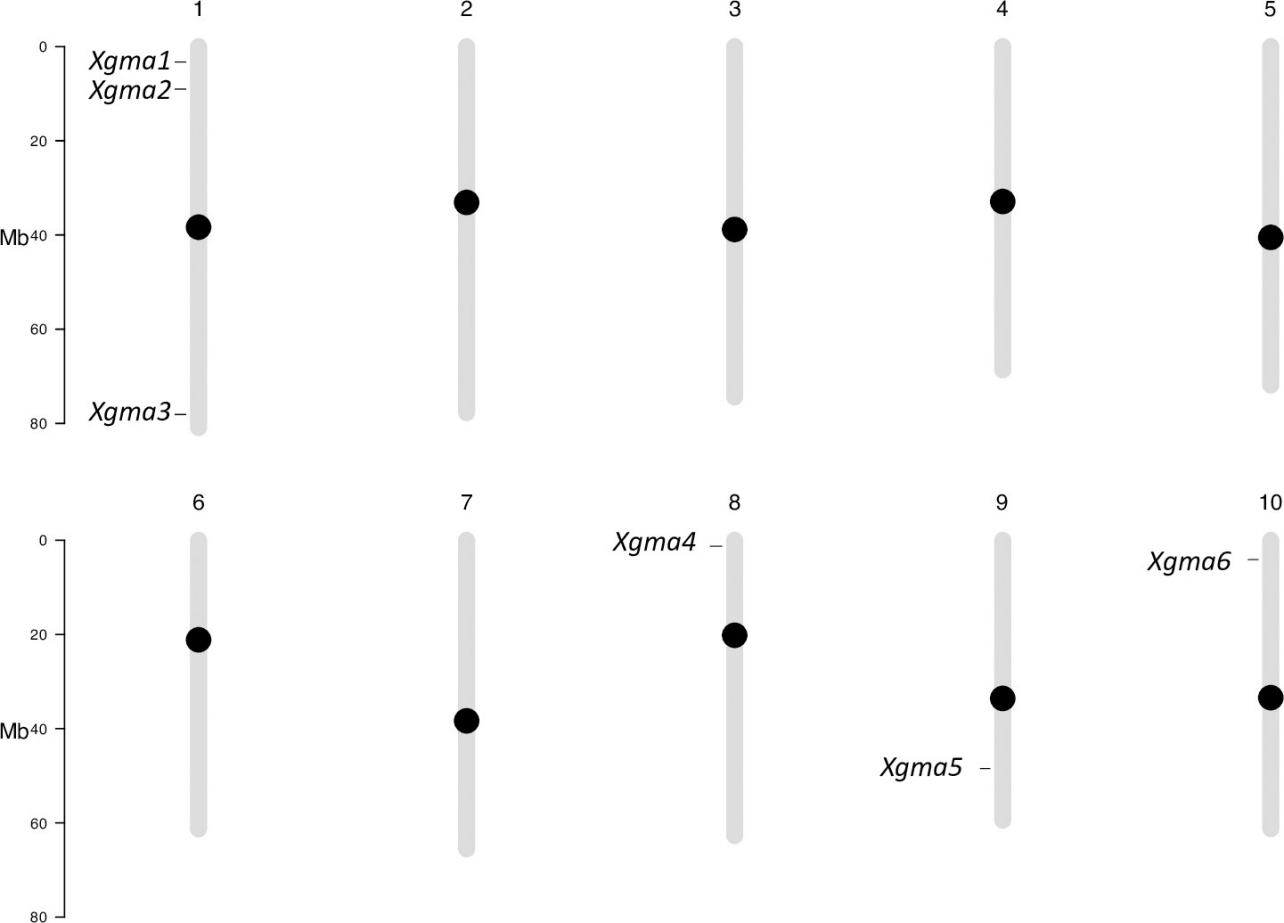
Chromosomal map of the six SSRs specific to margaritiferum. The ten chromosomes of *Sorghum bicolor* are represented in sequential order and labeled with the chromosome number above each. The bar on the left of each set of five chromosomes depicts the length in millions of bases (Mb). The black circles indicate centromeric regions. Each SSR is named and marked at its location on the chromosome.

The PI (Plant Introduction) numbers and accession name labels of the 53 accessions used for NGS validation of SSRs are provided in S1 Table. After seedling emergence, genomic DNA from the accessions was used in Illumina® directed paired end DNA NGS to amplify the 192 SSR sites. Sequencing summary statistics are provided in S2 Table. Of the 192 SSR sites 187 were successfully sequenced in the accessions analyzed in this manuscript. One SSR (Xunr43) only amplified in wild sorghum accessions included in sequencing run for a separate analysis, and six of the sites were unique for margaritiferum (S3 Table). These six SSRs are shown mapped to their chromosomal locations in Fig 4. The SSRs are denoted first with *X* to signify that they are SSRs, then with *gma* to identify their use for determining the guinea working group margaritiferum, and finally numbered one through six in order of chromosomal and genomic location starting from the first base pair on Chromosome one to the final base pair on Chromosome ten based on the current *Sorghum bicolor* reference genome [25]. The six SSRs, *Xgma1 –Xgma6*, their genomic locations, repeat sequences in the reference genome, repeat sequences specific to margaritiferum, as well as their corresponding forward and reverse primers, are shown in Table 1. The sequences of *Xgma1 –Xgma6* for the 53 accessions sequenced are presented in S3 Table. The rest of the SSRs identified are denoted as *X* to signify them as SSRs, then with *unr* to identify the location of their development (University of Nevada, Reno), and numbered 1–182 by the same ordination scheme of genomic location (S4 Table) as was used for *Xgma1 –Xgma6*. Based off the thresholds set by Botstein et al. 1980 [62], 114 of the SSRs presented here are highly informative (PIC > 0.5), 43 are reasonably informative (0.5 > PIC > 0.25), and 30 are slightly informative (PIC < 0.25). It is important to note that the single SSR (*Xunr43*) that amplified only in wild sorghum relatives sequenced for a separate analysis was not used to calculate PIC or H because it was not relevant to differentiating among sub-populations (S5 Table). In agreement with the observation that many SSR alleles are shared among non-margaritiferum accessions (S4 Table), common genetic structure was observed between accessions from the bicolor, caudatum, durra, and kafir sub-populations, whereas no shared genetic structure was found between accessions from the margaritiferum or guinea sub-populations when all variants were analyzed simultaneously (Fig 1). This finding is potentially the result of genetic admixture among bicolor, caudatum, durra, and kafir accessions in U.S. breeding programs, which have largely excluded guinea and margaritiferum. The genetic purity of margaritiferum accessions made it easy to distinguish from other sub-populations. Extensive admixture among accessions of other sub-populations reduced the number of doubletons that were unique to only one sub-population, and therefore the statistical power to generate unique SSR fingerprints for each one.

**Table 1:** NGS-validated primer sites and sequence lengths of margaritiferum-specific SSRs (*Xgma1—Xgma6*), based on the data set analyzed.

SSR Name	Chromosome	Start Location (bp)	End Location (bp)	Reference Repeat Structure	Repeat Structure in Margaritiferum	Forward Primer (+, 5’ to 3’))	Reverse Primer (-, 5’ to 3’)
***Xgma1***	1	3,295,349	3,295,385	GCG (12)	GCG (4)	TCGGTCGTGCCGGGAAGGGGGACTGGAGGGTAGGGTCTGG	CCCAACCGCAACAGACCAAACCCAGCCGCAACCGCAGAGC
***Xgma2***	1	9,033,349	9,033,373	GCC (8)	GCC (10)	GGTCGAGGGCTCCTCTCTCTCTACCGTCCCCGCTCGATCC	GGCAGCGCGACGATAGGCGCGAGTTGGACTGGCGGAGAGC
***Xgma3***	1	78,120,648	78,120,675	ACT (9)	ACT (6)	ATACATACATATCTGTTAGGCCATGCATGAACATCTAAAC	GAAACCGGAAGAAAGATTGCACTCGATCGTGTAATAGTCG
***Xgma4***	8	1,256,427	1,256,444	CCACGC (3)	CCACGC (2)	GCCCAGCAGACCAGCCGCGTTGGCTGGCGGGACGGAGCAG	CAGTAGACCGTCGGGACGGCGCCGCTGGGCCTCGGCTTGG
***Xgma5***	9	48,460,761	48,460,787	GGATG (5)	GGATG (4)	TGTGGGATTTCGCTTCTCGAGGGAACGGAATTACGGGAAG	GCCAAATCAAAGCTAGACTCGACGCTAGTGCCATGTGACG
***Xgma6***	10	4,107,816	4,107,841	GAG (8)	GAG (5)	GCCGGTGGTGGAGCGGTTGCGGCGGCGGCAGGGCACGGCC	CCACCCACTTGCCCCACTTGCGCATCCGAACGCCGCGATA

SSR names and genomic locations are listed. Repeat structure with repeat quantity, rounded to whole repeat, in parentheses for both the reference genome sequence and margaritiferum individuals are shown. The forward and reverse primers corresponding to each SSR are provided in 5’ to 3’ orientation for the + and—strands.

The numbers of repeats in *Xgma1 –Xgma6* are highly unique to margaritiferum accessions. For example, the *Xgma2* allele in margaritiferum accessions was ten (GCC) repeats, but no *Xgma2* alleles of this length were observed in non-margaritiferum accessions (S3 Table). *Xgma4* alleles in one margaritiferum accession did not produce sequencing data, but all others were homozygous for two (CCACGC) repeats; whereas no *Xgma4* alleles of this length were observed in non-margaritiferum accessions (S3 Table). The *Xgma6* allele in margaritiferum accessions had five (GAG) repeats and all accessions were homozygous for this length (S3 Table), whereas no *Xgma6* alleles of this length were observed in non-margaritiferum accessions. In combination, the length haplotypes amplified by our novel six SSRs primer sets (*Xgma1 –Xgma6*) can clearly differentiate the margaritiferum accessions from all other sub-populations that contributed to this analysis.

## Discussion

Other researchers have observed that there are likely more than five primary sub-populations within cultivated sorghum [5,6]. Depending on the number and ancestry of the accessions included in any population structure analysis, the distribution of populations may vary. The current project identified six distinct sub-populations (Figs 1 and 2) via genetic structure analysis and independently by the split network method among the 75 published WGS data sets in sorghum that were used for this analysis. The LLBO curve (S2 Fig), also denoting six as the optimal population size (*K*) for the sampled accessions, validated these findings. *In silico* analysis identified 163,943 SSRs in the *Sorghum bicolor* reference genome. Of those, 130,120 were present in 75 published WGS data sets and selected for further investigation based on read depth and sequence quality. While the six sub-populations identified shared many SSRs (65,512), each sub-population had 318 to 1,071 SSRs that were present in only that sub-population in the analyzed dataset (Fig 3). Nevertheless, one must be cautious interpreting this result, as conclusive fingerprinting of sub-population margaritiferum was likely only possible because it was free of admixture. Larger sample sizes representing the remaining five sub-populations are needed for WGS, particularly accessions with minimal admixture from other sub-populations, in order to develop SSR fingerprints with the greatest ability to assess population structure in sorghum and contribute the most meaningful genetic diversity information to breeding for yield gains through improved heterosis, as has been done in wheat and maize [42,43].

3,334 SSRs were predicted by HipSTR from the *Sorghum bicolor* reference genome that were not found in any of the merged VCF files from the 75 accessions studied at those locations. This is possibly an effect of differing sequencing depths among accessions used in file merging or SSRs that only exist in the individual used for the reference genome. Within the 130,120 SSRs identified in the 75 accessions studied, there were 4,179 unique doubleton locations that were filtered using the methods described to narrow down the NGS validation set to 192. These SSR sites were investigated for sub-population determination among all 53 publicly-available sorghum accessions available from Grin-Global [59] at the time of this project. The unique method doubleton filtering, compared to other SSR-NGS sequencing projects [38–41], was the key to enriching for SSRs that efficiently identified population structure. Doubleton filtering is better at identifying unique alleles specific to an entire sub-population whereas singleton filtering is better suited to identifying rare alleles within sub-populations. Doubleton filtering to identify sub-populations was achieved by merging accessions within each sub-population prior to analysis of the WGS data, essentially treating the merged file as one individual versus using each accession as its own individual.

Of the 192 genomic locations selected for NGS validation, six unambiguously differentiated margaritiferum accessions from bicolor, caudatum, durra, guinea, and kafir accessions, making them ideal genetic diversity markers for this purpose. These six SSRs, mapped in Fig 4 and described in Table 1, can be used to identify margaritiferum individuals in the analyzed data sets. Additional SSRs may be tested and validated using the data sets provided. The sequences of *Xgma1 –Xgma6* (S3 Table) show how a simple Excel output file allows SSRs to be easily visually compared among sub-populations, thus providing accessible genetic diversity information to students and researchers with limited bioinformatics training.

DNA markers have long been used to study genetic relatedness in plants and forensics in humans. Along with SSRs other commonly used markers of genetic analysis include restriction fragment length polymorphisms (RFLPs), DArT markers, and SNPs. SSRs have advantages of increased power of discrimination among sub-populations, are more reliable, and have better repeatability than RFLPs [67] and SNPs [18], and are more modular and customizable in format than DArT markers, which come in sets and rely on solid-state sequencing platforms. Nevertheless, SSR sequencing has historically been cumbersome and fell out of favor once GBS-SNP pipelines were developed. Unfortunately, a selection bias is introduced by using the direct identification of genomic regions responsible for desired phenotypes to form the basis of estimating genetic diversity, instead of molecular markers that may or may not be linked to traits under artificial selection [18]. It is worth investigating whether or not cost savings can be introduced in GS pipelines currently relying on GBS-SNPs by diversifying marker types to reduce the total number of loci sequenced. For example, NGS-SSR markers could be used to assess the genetic background of entire genomes or even specific chromosomal regions, as well as the genetic diversity of breeding lines; and SNP markers could be used in parallel to focus on functional phenotypic variation. The methods outlined herein are ideal for developing sets of SSRs specific to the germplasm and needs of individual breeding programs that could be used to test this strategy.

The availability of NGS-SSR pipelines enables the evaluation of genetic diversity at a throughput not previously possible, and they can be deployed in any species with a reference genome. NGS techniques are currently powerful enough that even genetically “identical” twins can be differentiated from one another [68]. The power of NGS coupled with the low cost of targeted sequencing makes methodologies like those described in this paper an exciting frontier in population genetics, heterotic group development in breeding programs, and the genetic identification of patented materials.

## Conclusions

Our results divided sorghum into six distinct sub-populations (bicolor, caudatum, durra, guinea, kafir, and margaritiferum) based both on genetic structure and split network mapping analyses of 75 published WGS data sets and comparisons with the *Sorghum bicolor* reference genome. To help identify margaritiferum from the remaining sub-populations, six novel SSR primer sets (*Xgma1 –Xgma6*) suited for targeted NGS are presented. 182 additional novel SSR primer sets (*Xunr1 –Xunr182*) are presented and may be used to develop similar fingerprints for the remaining sub-populations once more WGS information for each one becomes available. These SSRs are reported with their repeat motifs, the quantity of repeats in the reference genome and (where applicable) in margaritiferum accessions; forward and reverse primer sequences, and PIC and H values. This project demonstrates the amenability of SSRs to targeted sequencing and NGS and lays out a framework for future work standardizing molecular characterizations of sub-populations in sorghum.

## Supporting information

S1 Fig Accessions’ genetic structure of *K* = 2–10 shows 6 is the maximum population size. FastSRTUCTURE plotting in ggplot2 of accessions’ genetic structure from *K* equals 2 to 10 in order. Color depicting each population (P1-P10) shown in the corresponding legend on the right of each graph.
(PDF)

S2 Fig Marginal likelihood identifies 6 as the optimum *K* value. The log-marginal likelihood lower bound (*y*-axis) calculated in fastSTRUCTURE and plotted against the *K* population size (*x*-axis) shows 6 is the optimum population size to maximize the marginal likelihood. Dashed line drawn at -.709 marginal likelihood for cut off between *K* = 5 & 7 versus *K* = 6.
(PDF)

S1 Table Accessions sequenced using NGS. This table includes the accession names and PI numbers that were grown and used for NGS sequencing.
(XLSX)

S2 Table Read count table for 53 sequence accessions on Illumina® NextSeq 550. The read count table is presented for all of the samples sequenced by NGS.
(XLSX)

S3 Table Sequences of accessions based on SSR. This table includes the sequencing data for each of the 53 accessions at *Xgma1 –Xgma6*. The accessions are sorted based on population. The reference allele and all alternative alleles for the SSRs are included at the bottom of the table.
(XLSX)

S4 Table Sequences of all SSRs (*Xgma1 –Xgma6* & *Xunr1 –Xunr182*). This table includes the sequencing data of the 53 accessions at *Xgma1 –Xgma6* & *Xunr1 –Xunr182*. The chromosomal locations of the SSRs, the primers (forward and reverse) with their associated Tms, and the reference genome sequence are provided.
(XLSX)

S5 Table PIC and H values of all SSRs (*Xgma1 –Xgma6* & *Xunr1 –Xunr182*). This table includes the PIC and H values calculated for *Xgma1 –Xgma6* & *Xunr1 –Xunr182* based on the 53 accessions sequencing data.
(XLSX)
