# Supplementary figures and images for: De novo identification and targeted sequencing of SSRs efficiently fingerprints Sorghum bicolor sub-population identity
Source: PLoS One. 2021 Mar 8;16(3):e0248213. doi: 10.1371/journal.pone.0248213 (PMC7939377; doi:10.1371/journal.pone.0248213)

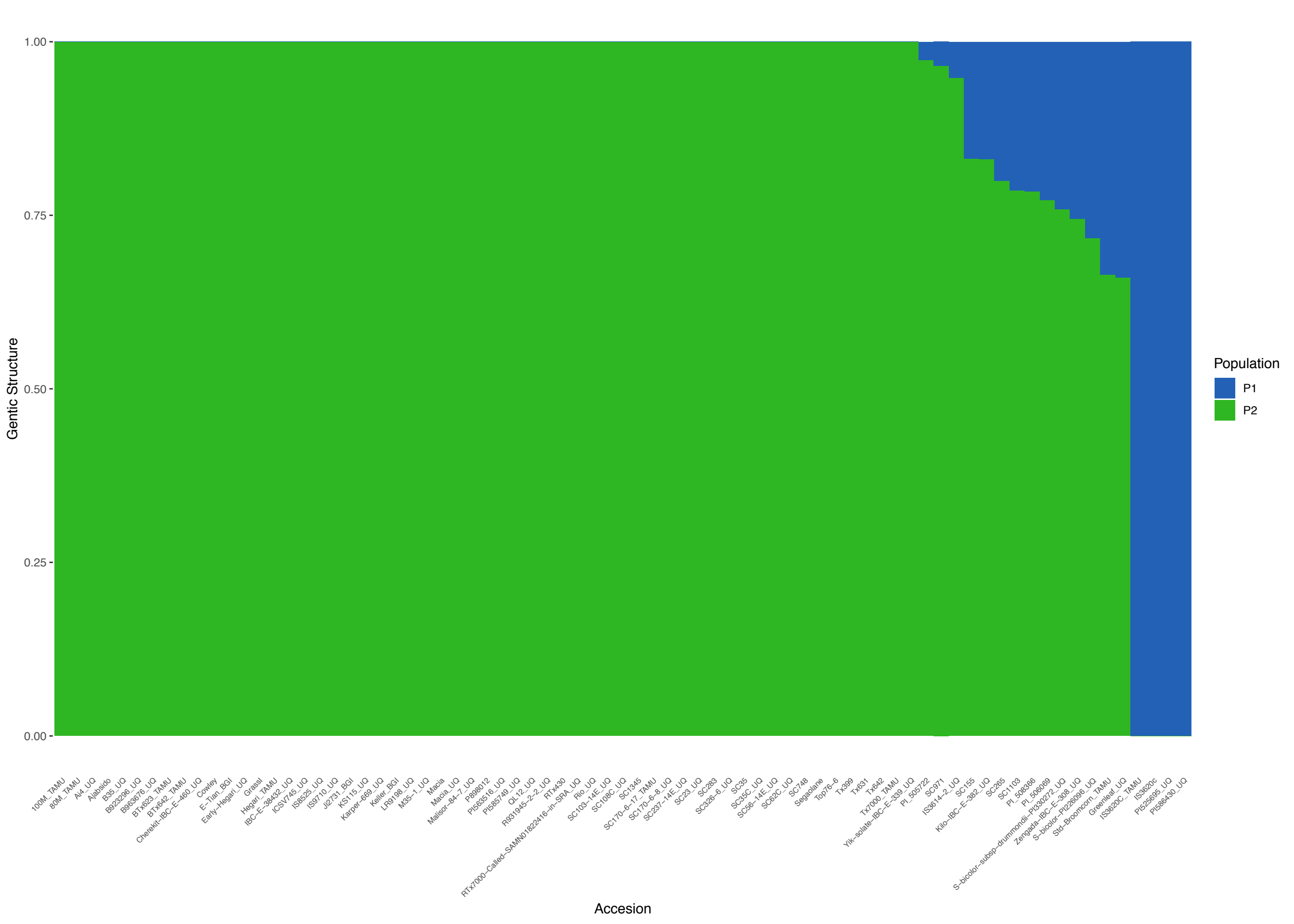

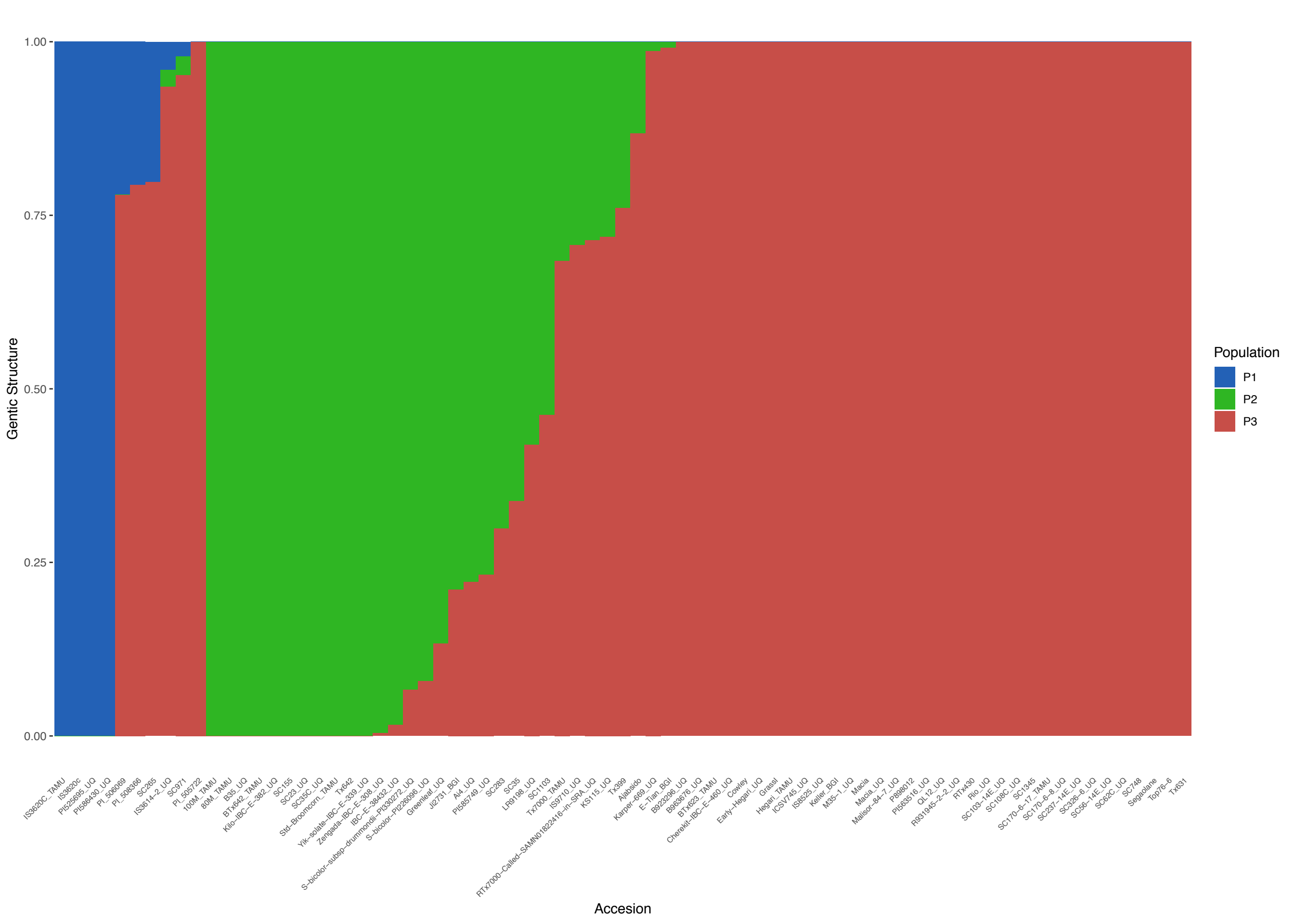

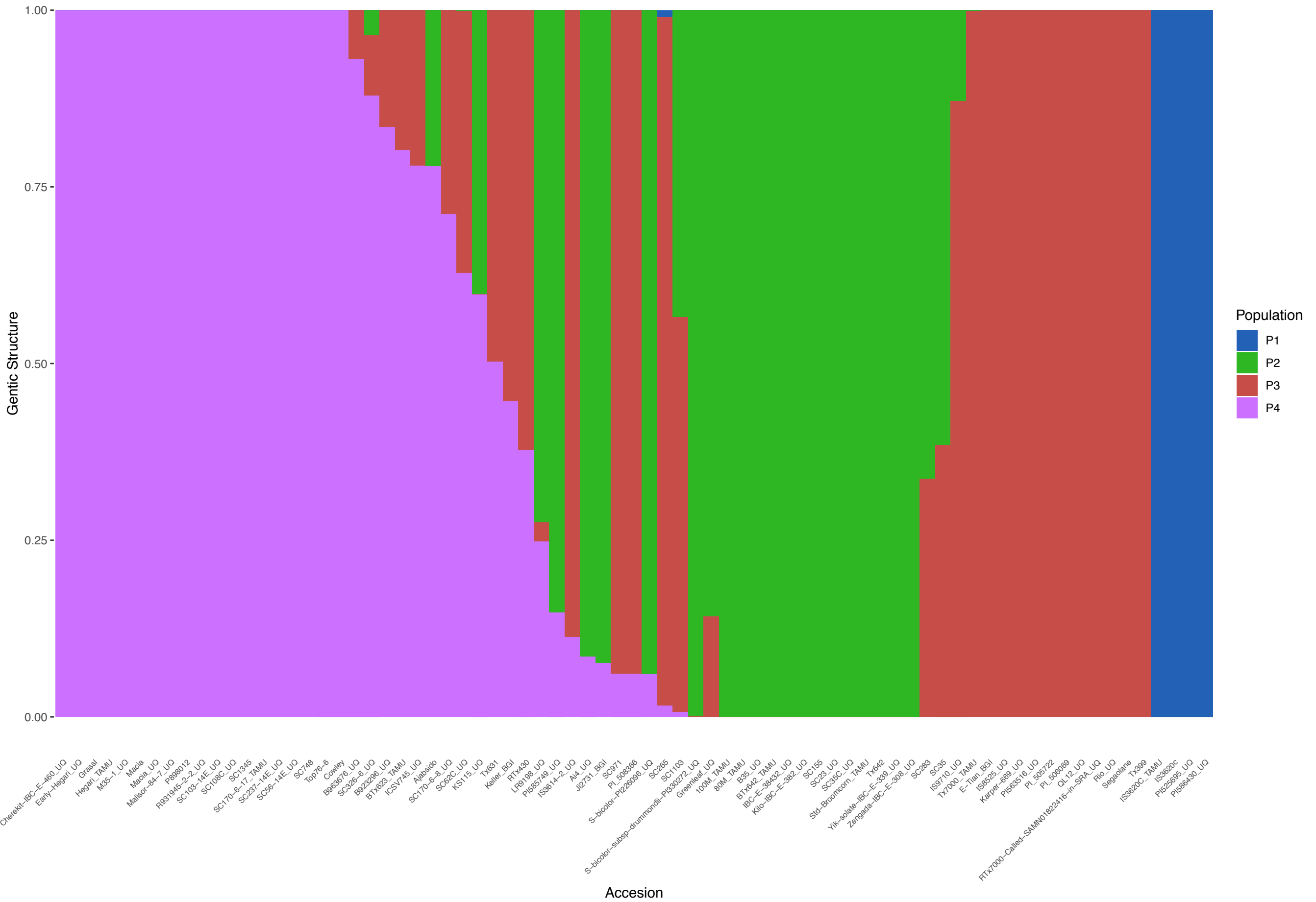

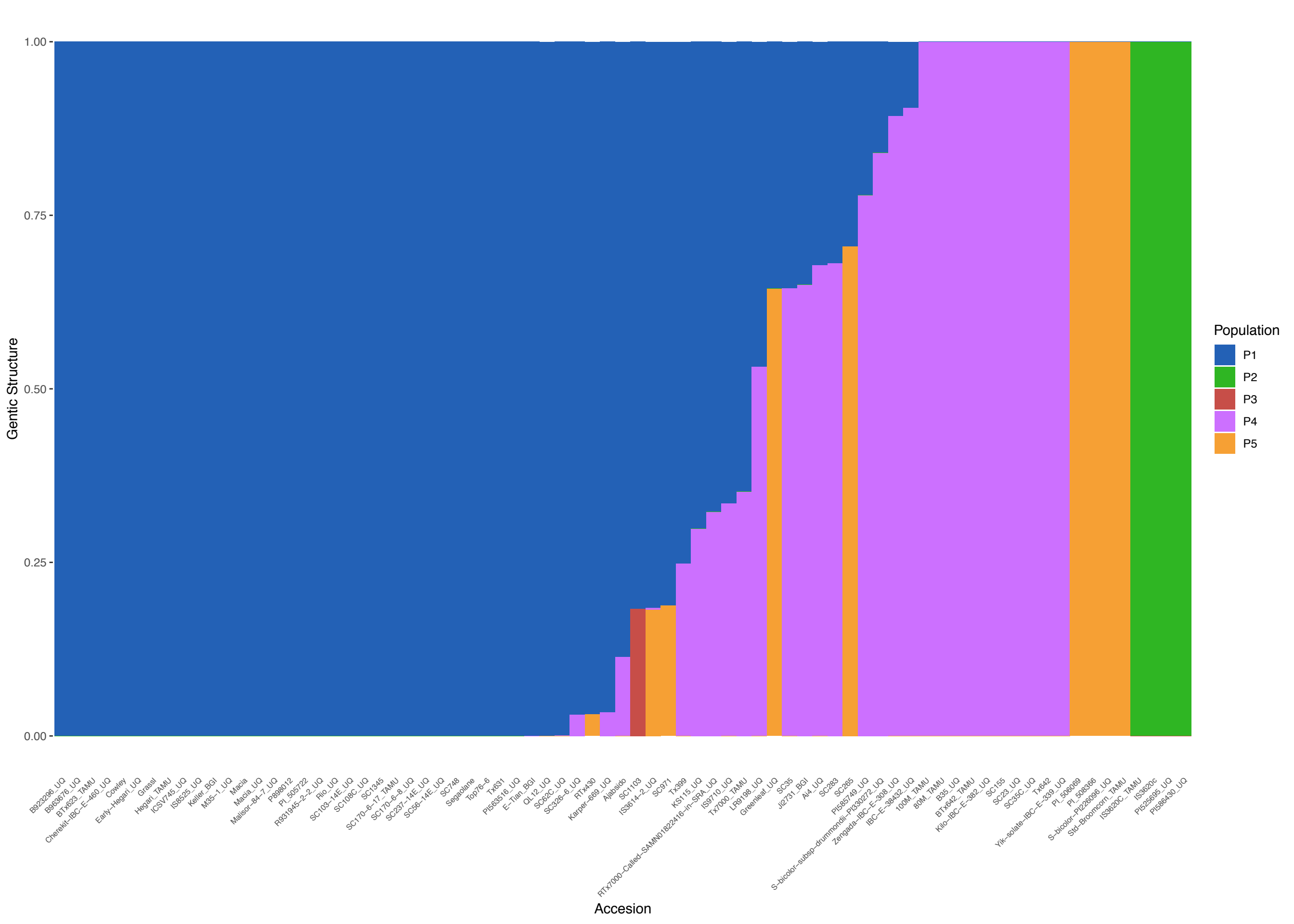

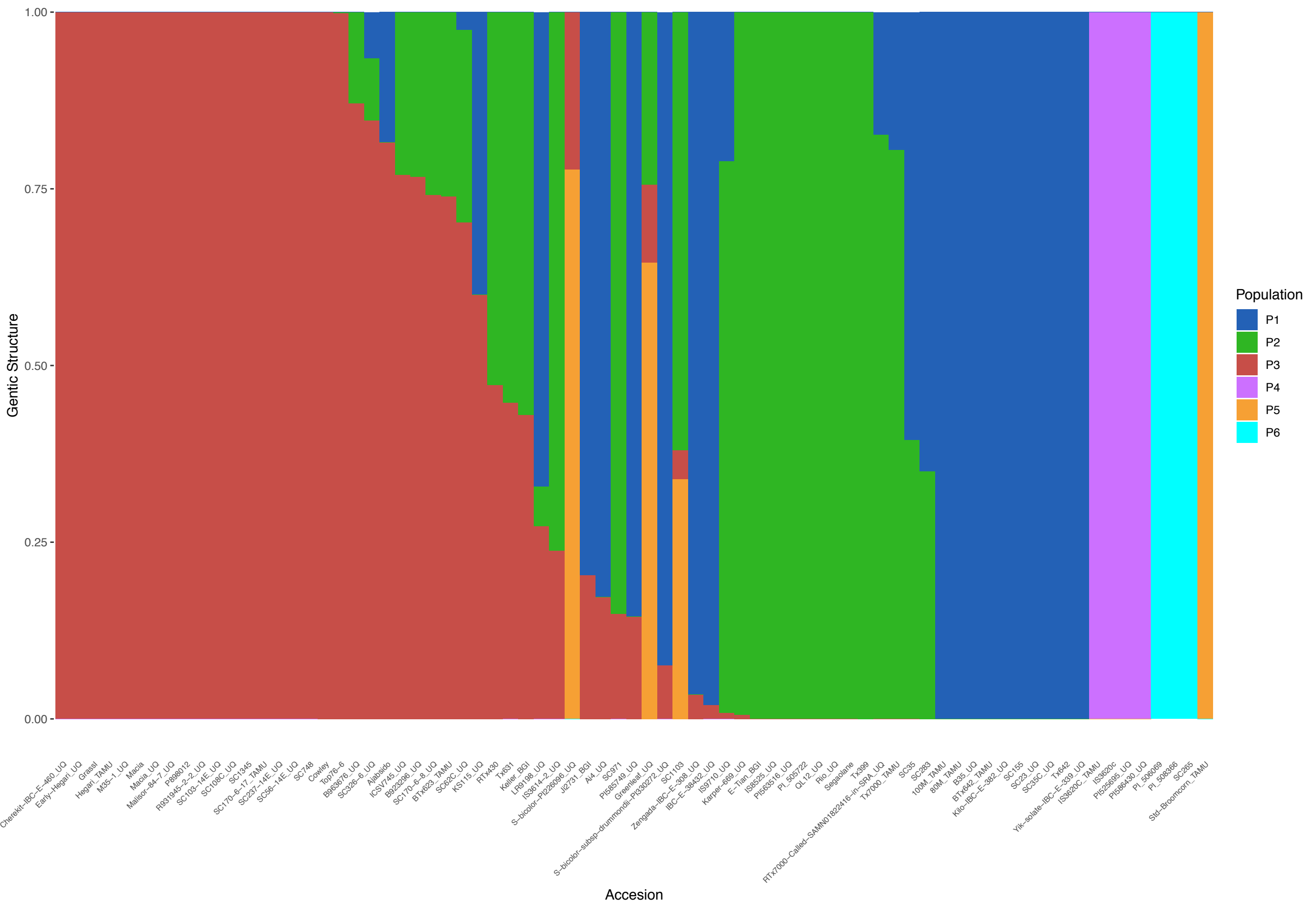

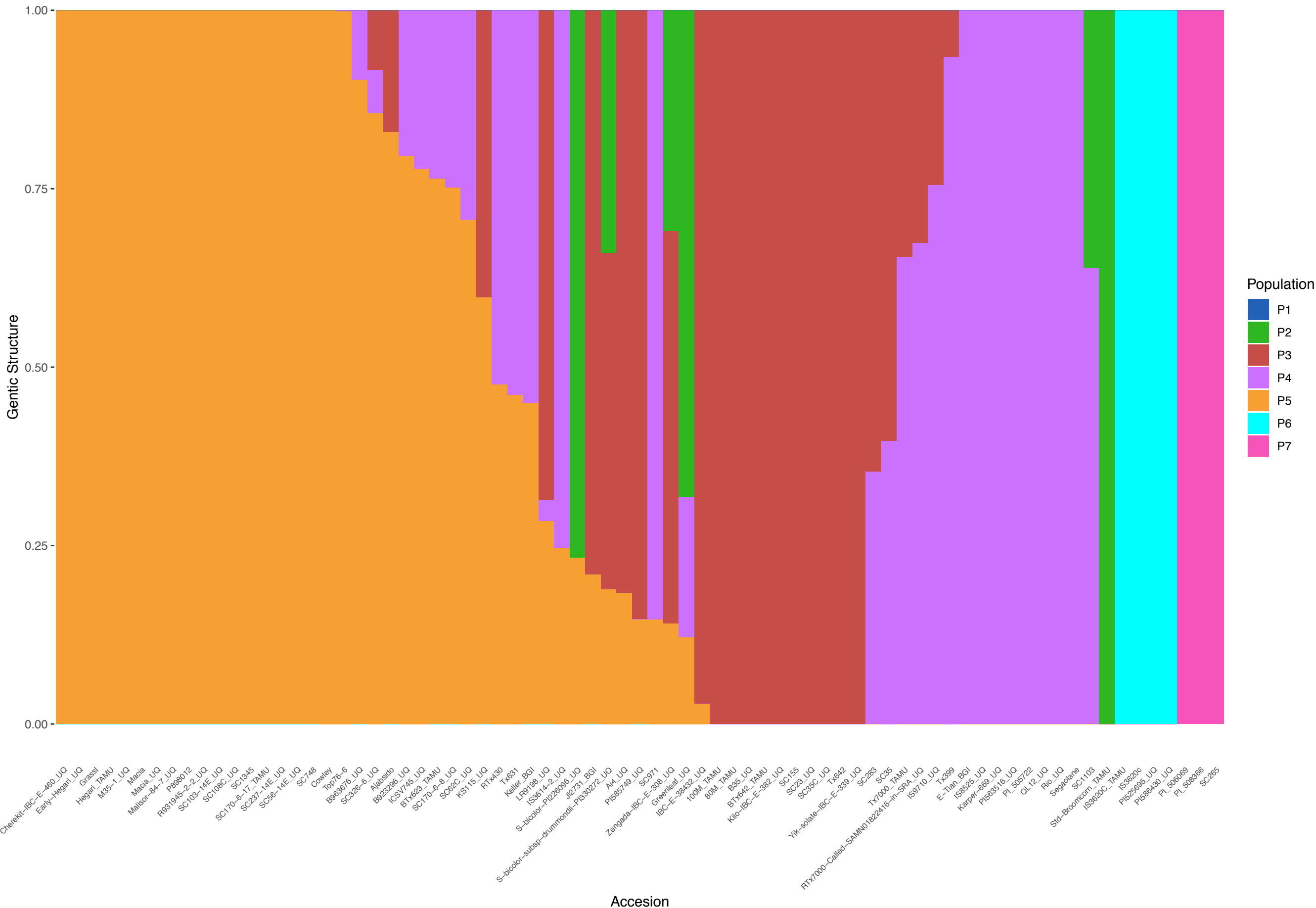

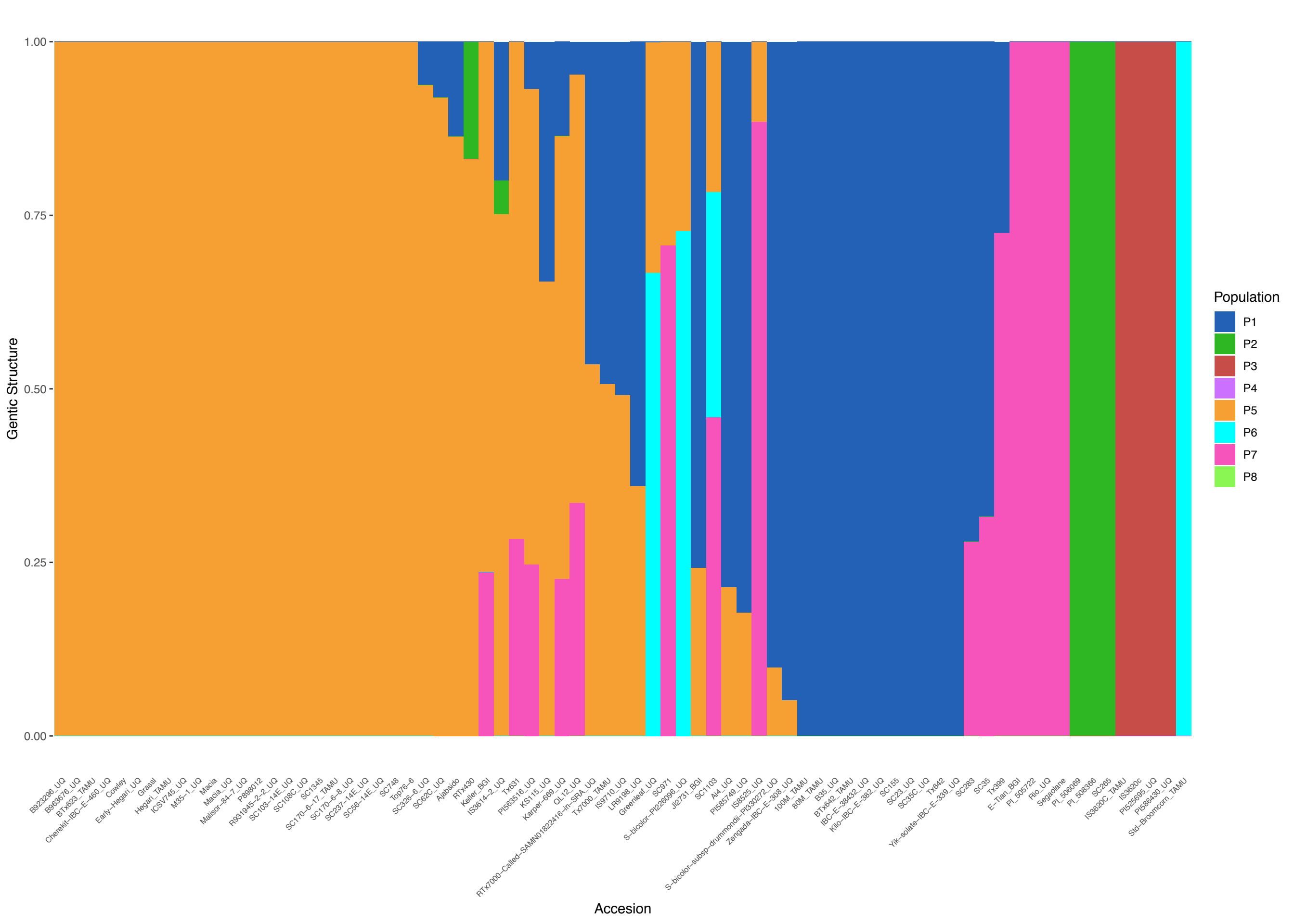

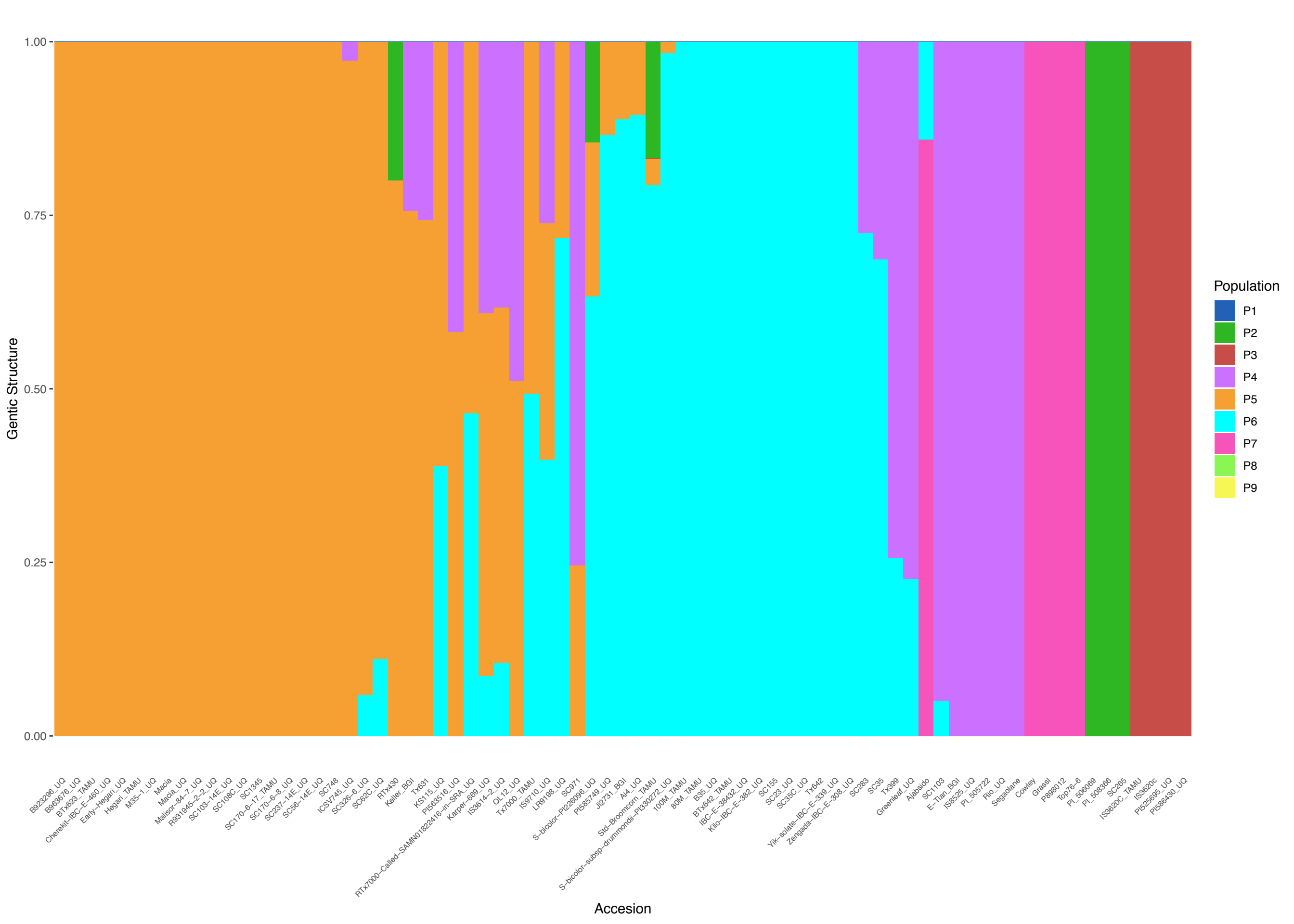

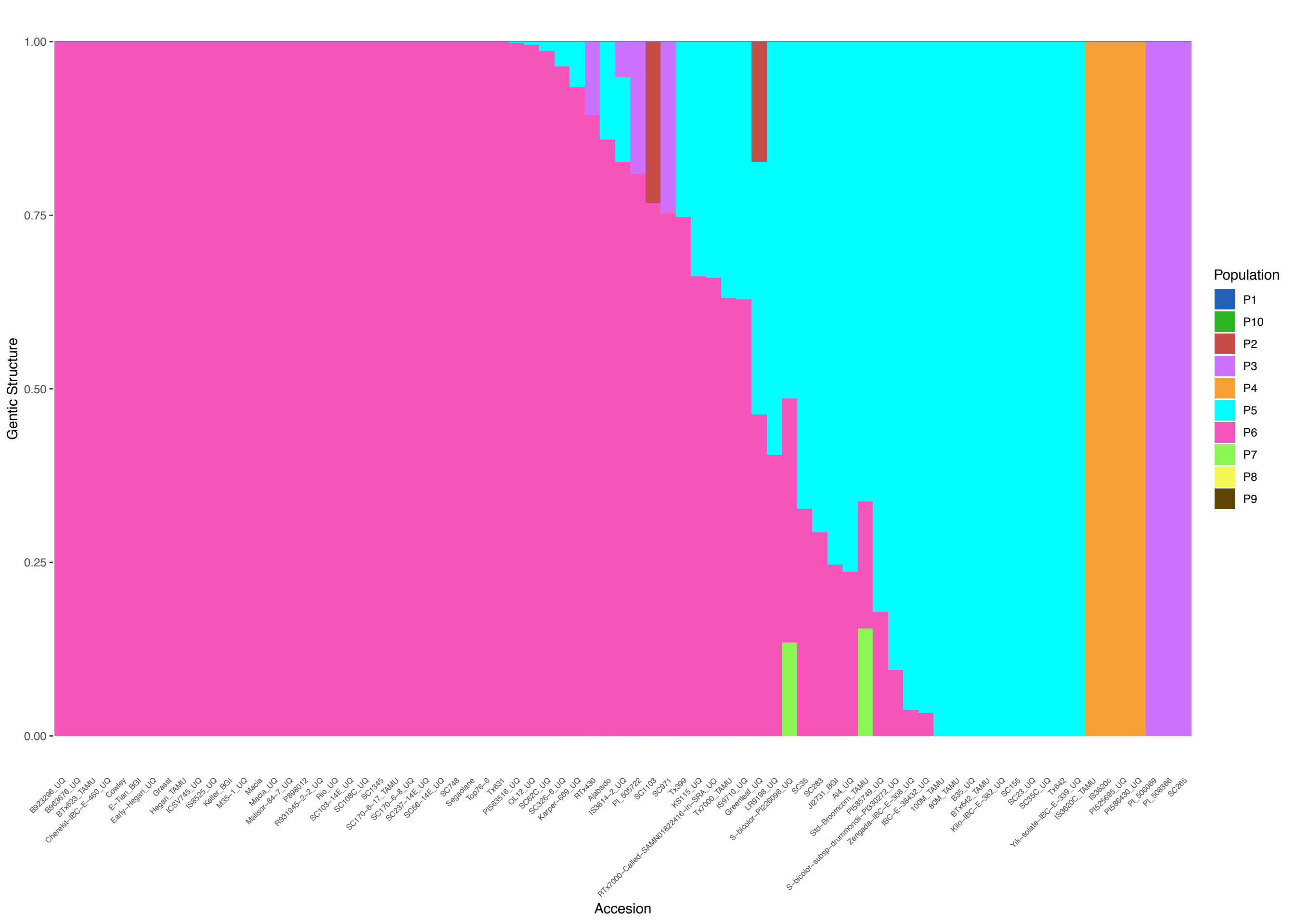

Supplement: S1 Fig — FastSRTUCTURE plotting in ggplot2 of accessions’ genetic structure from K equals 2 to 10 in order. Color depicting each population (P1-P10) shown in the corresponding legend on the right of each graph. (PDF) [file pone.0248213.s001.pdf]

Marginal Likelihood

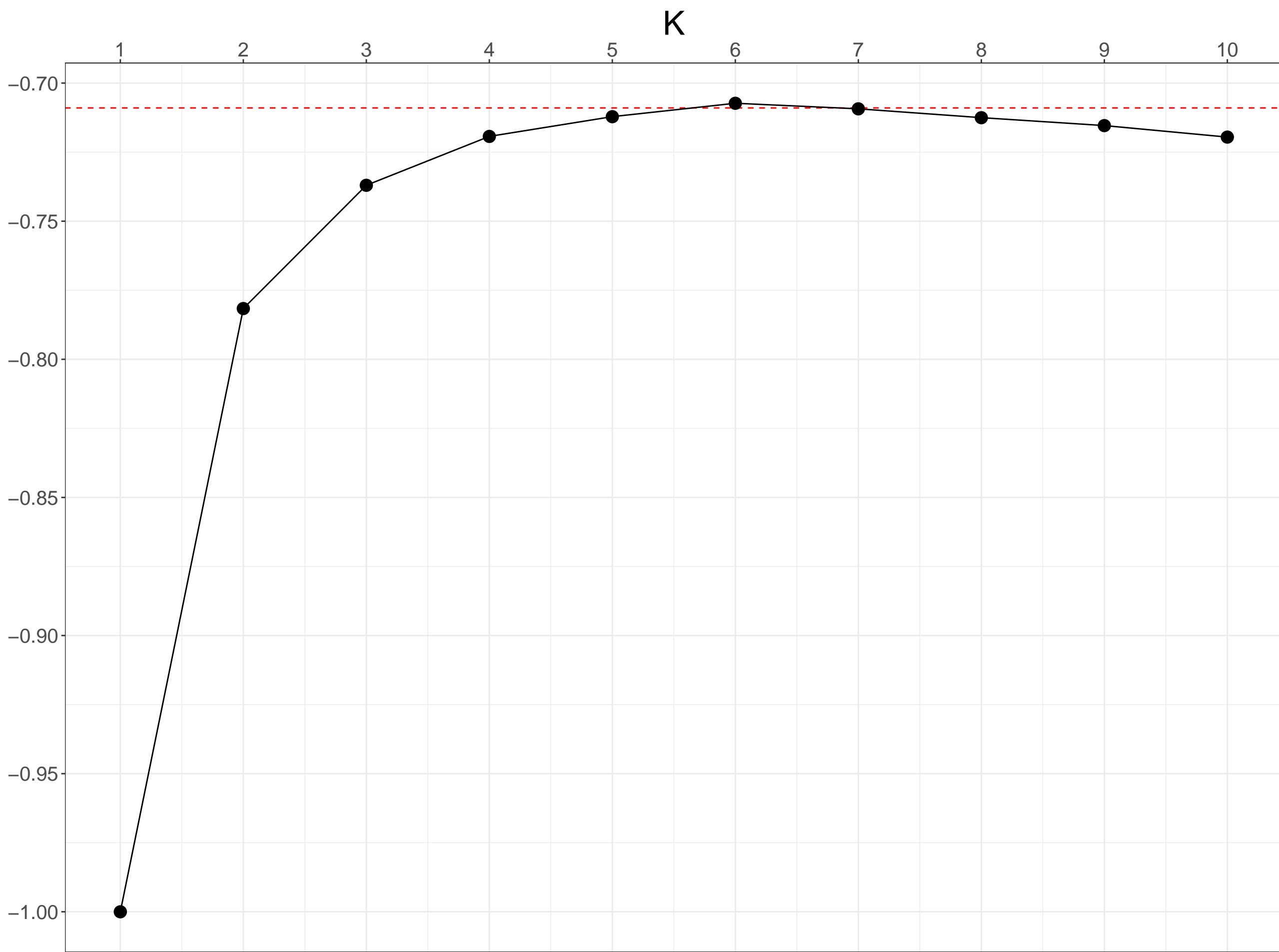

Supplement: S2 Fig — The log-marginal likelihood lower bound (y-axis) calculated in fastSTRUCTURE and plotted against the K population size (x-axis) shows 6 is the optimum population size to maximize the marginal likelihood. Dashed line drawn at -.709 marginal likelihood for cut off between K = 5 & 7 versus K = 6. (PDF) [file pone.0248213.s002.pdf]
